# Adenosine Monophosphate as a Metabolic Adjuvant Enhances Antibiotic Efficacy against Drug-Resistant Bacterial Pathogens

**DOI:** 10.3390/ph17070933

**Published:** 2024-07-11

**Authors:** Wenxuan Zhang, Zhenyi Wu, Zulifukeer Maituersong, Ting Wang, Yubin Su

**Affiliations:** Department of Cell Biology & Institute of Biomedicine, National Engineering Research Center of Genetic Medicine, MOE Key Laboratory of Tumor Molecular Biology, Guangdong Provincial Key Laboratory of Bioengineering Medicine, College of Life Science and Technology, Jinan University, Guangzhou 510632, China; zhangwenxuan@stu2021.jnu.edu.cn (W.Z.); wzhenyi2024@stu2021.jnu.edu.cn (Z.W.); zulpikar@stu2022.jnu.edu.cn (Z.M.); wangting1988@jnu.edu.cn (T.W.)

**Keywords:** antimicrobial resistance, gentamicin-resistant *Staphylococcus aureus*, adenosine monophosphate, gentamicin

## Abstract

Global bacterial infections are on the rise, and drug resistance to bacteria is gradually rendering existing antibiotics ineffective. Therefore, the discovery of new strategies is urgently needed. Cellular metabolism is a key factor in the regulation of bacterial drug resistance, which cannot be separated from the utilization of energetic substances, suggesting that energetic substances may be associated with bacterial drug resistance. In this study, we found that adenosine monophosphate (AMP) can enhance the bactericidal effect of gentamicin against gentamicin-resistant *Staphylococcus aureus.* This synergistic effect can be generalized for use with different antibiotics and Gram-positive or Gram-negative bacteria. We also validated that the mechanism of AMP reversal of antibiotic resistance involves enhancing the proton motive force via the tricarboxylic acid cycle to increase antibiotic uptake. Simultaneously, AMP increases oxidative stress-induced cell death. This study presents a strategy for adopting low-dose antibiotics to control drug-resistant bacteria, which is important for future drug development and bacterial control.

## 1. Introduction

With the widespread use of antibiotics, many bacteria have developed resistance to multiple antibiotics, making the control of many otherwise treatable diseases difficult [1]. Antibiotic resistance is projected to cause up to 10 million deaths annually by 2050 if bacterial resistance is not controlled or improved [2]. Therefore, an in-depth study of bacterial drug resistance mechanisms and the search for new therapeutic strategies have become current hotspots in biomedical research.

In view of the currently growing drug resistance, the WHO has listed six categories (referred to as ESKAPE) of conditionally pathogenic bacteria that need to be prioritized for eradication [3]: *Enterococcus faecalis*, *Staphylococcus aureus*, *Klebsiella pneumoniae*, *Acinetobacter baumannii*, *Pseudomonas aeruginosa*, and a series of *Enterobacteriaceae*. Among the six categories, *S. aureus* is the only Gram-positive bacterium. It is a major pathogen in nosocomial and community-acquired infections and has become a serious health problem [4]. *S. aureus* can induce various infections in different parts of the body, including abscesses, endocarditis, and osteomyelitis [5]. Today, the use of antibiotics, such as daptomycin and vancomycin, remains the mainstay of treatment for *S. aureus* infections [5,6]; however, as overuse of antibiotics has gradually led to the development of broad-spectrum resistance in bacteria [7,8], the development of effective treatment options is urgently needed.

Currently, different types of antibiotics that mediate different pathways are used to treat *S. aureus*. Antibiotics such as β-lactams, tetracyclines, and sulfonamides treat *S. aureus* infections via mechanisms acting on cell membranes, protein synthesis, and DNA replication [9]. Aminoglycosides, especially gentamicin, are the preferred class of drugs because of their broad-spectrum antimicrobial and rapid bactericidal properties, which enter the intracellular compartment through a proton motive force [10]. Upon entering the cell, gentamicin usually binds to the 30S ribosome to interfere with bacterial protein synthesis, resulting in the production of mistranslated proteins and, ultimately, cell death [11,12]. However, with the emergence of drug-resistant strains, the efficacy of gentamicin is seriously threatened.

In response to traditional drugs and treatments becoming ineffective, researchers are attempting to devise new strategies in the field of antibiotics, such as polymyxins and their derivatives [13] and natural phytochemicals [14]. However, despite good experimental and clinical performance of these new drugs as alternatives to conventional antibiotics, they suffer from a number of problems, such as long development cycles, high investment, high risk, and the possibility that the long-term use of new antibiotics may lead to bacterial resistance. To solve this problem, constant updates to our thinking and the discovery of ways to improve the therapeutic efficiency of existing antibiotics are critically needed to achieve “new use of old drugs” and to reduce the emergence of bacterial resistance.

Several studies have investigated the effects of exogenous substances on bacterial resistance. Some studies have reprogrammed bacterial metabolism with substances such as nucleotides and amino acids, especially increasing the tricarboxylic acid (TCA) cycle or pyruvate (P) cycle, to promote bacterial sensitivity to antibiotics [15,16]. In addition, sugars such as glucose and fructose allow more antibiotics to enter the cell by promoting proton motive force (PMF) [17,18]. These substances regulate bacterial metabolic pathways, reverse bacterial resistance, and enable the use of old drugs for new purposes [19]. Various bacterial metabolic pathways are involved in the recycling of energy substances. This association between metabolic regulation and energy sources provides new perspectives for understanding and solving the problem of bacterial drug resistance [20,21]. However, most of the proposed bactericidal effects have been applied to Gram-negative bacteria, such as *Escherichia coli* and *K. pneumoniae*; in contrast, Gram-positive bacteria have been less studied. In addition, whether the incorporation of exogenous energy substances contributes to the enhancement of antibiotic-mediated killing and its mechanism of action remain unexplored.

In the present study, we explore energetic substances such as adenosine monophosphate (AMP) etc. to reverse antibiotic resistance in gentamicin-resistant *S. aureus*. We further demonstrate the synergistic killing with gentamicin plus AMP of other Gram-negative ESKAPE pathogens. The mechanism of AMP-enabled bacteria killing is related to the TCA cycle, which can drive proton motive force, and thus, elevate intracellular gentamicin levels and enhance bactericidal effects. Furthermore, AMP reduces the intracellular adenosine 5′-triphosphate levels, which associated with a futile cycle, and increases the reactive oxygen species levels. This study provides new evidence that AMP restores the sensitivity of bacterial pathogens to antibiotics.

## 2. Results

### 2.1. Exogenous AMP Promotes Gentamicin Killing of Gentamicin-Resistant S. aureus

**Figure 1:**
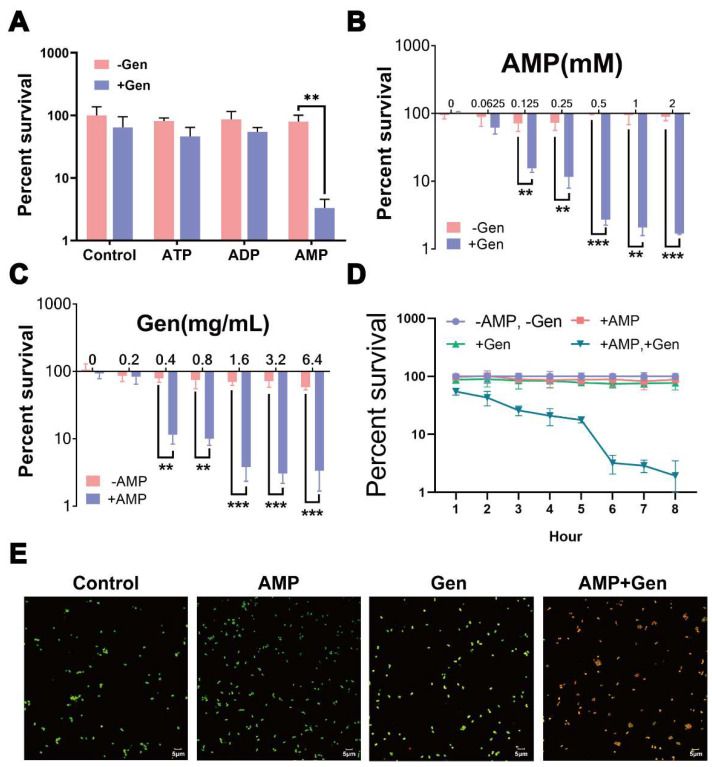
AMP promotes the killing of *S. aureus* with gentamicin. (**A**) Survival rate of gentamicin-resistant *S. aureus* treated with 3.2 mg/mL gentamicin and 10 mM ATP/ADP/AMP. (**B**) Survival rate of gentamicin-resistant *S. aureus* treated with different AMP concentrations of 1.6 mg/mL gentamicin. (**C**) Survival rate of gentamicin-resistant *S. aureus* treated with different concentrations of gentamicin 0.5 mM AMP. (**D**) Time effect of combined germicidal efficacy with and without 0.5 mM AMP and/or 1.6 mg/mL gentamicin. (**E**) Confocal micrographs of gentamicin-resistant *S. aureus*. Live bacteria were stained green with (9Z)-N,N-Dimethyl-9-octadecen-1-amine, while dead bacteria were stained red with EthD-III. Results are displayed as mean ± SEM, and significant differences are identified (** *p* < 0.01, *** *p* < 0.001) as determined using two-tailed Student’s *t*-test.

To better study bacterial drug resistance, we used *S. aureus* ATCC6538 as the initial strain and obtained gentamicin-resistant *S. aureus* via an artificial in vitro substitution culture; its minimum inhibitory concentration (MIC) was 128 folds higher than that of the initial strain [22]. To explore the synergistic bactericidal effects of energy substances with antibiotics, we observed that AMP had a synergistic effect with gentamicin, whereas ADP and ATP had no synergistic effects (Figure 1A). Therefore, we conducted an AMP assay. The use of AMP or gentamicin alone did not make a difference in bacterial survival; however, when used in combination, the killing effect was in the order of 100 folds higher, with 0.5 mM AMP and 1.6 mg/mL gentamicin (Figure 1B,C). The synergistic bactericidal effect of AMP with gentamicin was time-dependent, with the bactericidal effect stabilizing after 6 h of treatment (Figure 1D). Fluorescent fiber imaging showed that strains treated with only AMP or gentamicin were mostly green, whereas combined treatments resulted in enhanced orange versus red fluorescence, demonstrating that the bacteria appeared to be damaged to varying degrees (Figure 1E). These results indicate that AMP promotes the synergistic bactericidal properties of gentamicin against gentamicin-resistant *S. aureus.*

### 2.2. AMP Enhances Proton Motive Force via the TCA Cycle to Increase Antibiotic Uptake

**Figure 2:**
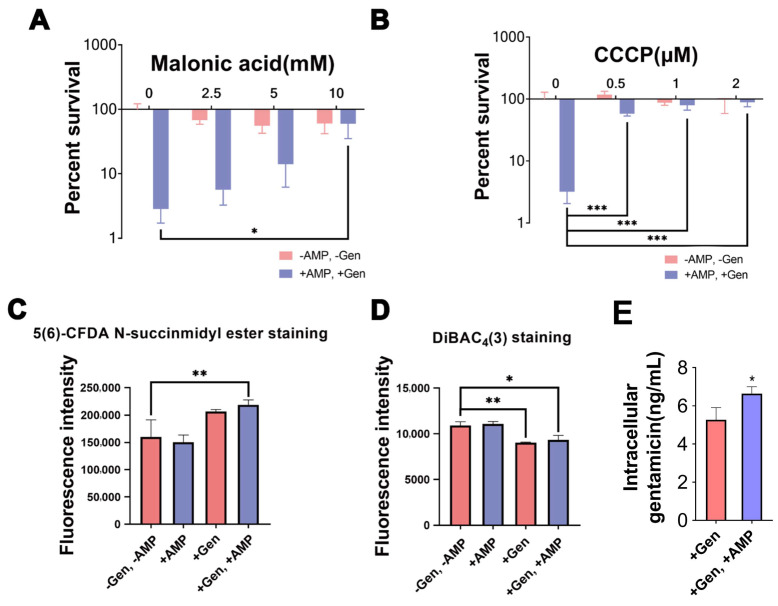
Exogenous AMP and gentamicin affect the TCA cycle and electron transport chain in gentamicin-resistant *S. aureus*. (**A**,**B**) Effect of malonic acid or CCCP concentration on germicidal efficacy of 1.6 mg/mL gentamicin plus 0.5 mM AMP treatment. (**C**,**D**) 5(6)-CFDA N-succinmidyl ester or DiBAC_4_(3) staining fluorescence intensity of gentamicin-resistant *S. aureus* treated with 1.6mg/mL gentamicin and/or 0.5 mM AMP. (**E**) Intracellular gentamicin content detected with 1.6 mg/mL gentamicin and/or 0.5 mM AMP. Results are displayed as mean ± SEM, and significant differences are identified (* *p* < 0.05, ** *p* < 0.01, *** *p* < 0.001) as determined using two-tailed Student’s *t*-test.

The TCA cycle and cellular respiration play important roles in bacterial drug resistance [15,20]. In this study, malonic acid (a competitive inhibitor of succinate dehydrogenase) also increased bacterial survival (Figure 2A). Subsequently, we found that the PMF inhibitor Carbonyl Cyanide3-ChloroPhenylhydrazone (CCCP) similarly increased the survival rates (Figure 2B). PMF enhanced the antimicrobial activity of gentamicin via pH gradients (ΔpH) or the membrane potential (Δψ), and the fluorescence intensity of 5(6)-CFDA N-succinmidyl ester and DiBAC_4_(3) staining were positively correlated with intracellular pH and membrane depolarization, respectively [23,24,25]. The results showed that ΔpH increased after combined treatment with gentamicin and AMP (Figure 2C). At the same time, the level of membrane depolarization decreased, indicating an increase in Δψ (Figure 2D). Exogenous AMP increased the intracellular gentamicin content in cells, as expected (Figure 2E). Therefore, the addition of gentamicin with AMP increased intracellular PMF. We suggest that the exogenous addition of AMP can drive PMF through the TCA cycle, elevate intracellular gentamicin levels, and enhance bactericidal effects.

### 2.3. AMP Induces Bacterial Oxidative Stress for a Synergistic Bactericidal Effect

**Figure 3:**
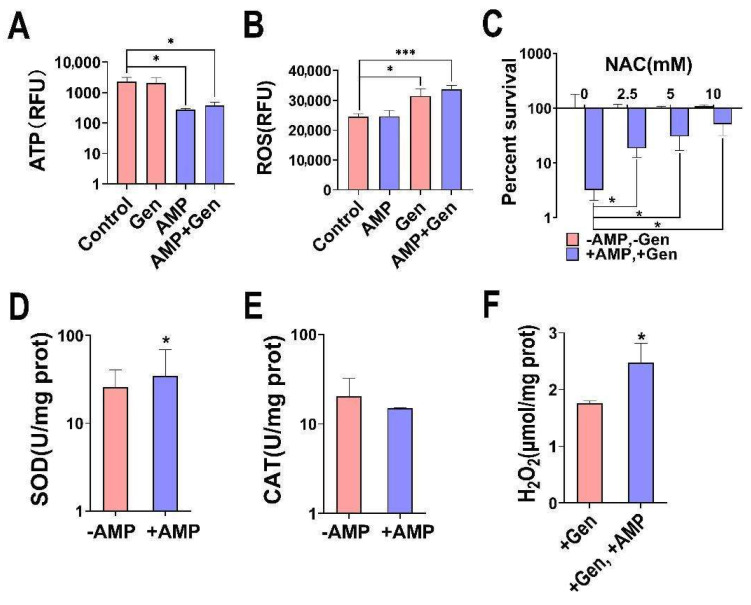
Synergistic bactericidal effect of AMP and gentamicin against gentamicin-resistant *S. aureus* in relation to ROS levels. (**A**,**B**) Determination of ATP and ROS. (**C**) Survival rate of gentamicin-resistant *S. aureus* when 1.6 mg/mL gentamicin and 0.5 mM AMP were incubated with different concentrations of NAC. (**D**–**F**) Determination of SOD, CAT, and H_2_O_2_. Results are displayed as mean ± SEM, and significant differences are identified (* *p* < 0.05, *** *p* < 0.001) as determined with two-tailed Student’s *t*-test.

The presence of intracellular reactive oxygen species (ROS) correlates with intracellular electron transport and antibiotic activity. Generally, the higher the level of ROS, the more effective antibiotics are at killing bacteria [26,27]. We observed that the addition of AMP alone reduced the intracellular ATP levels and increased the ROS levels in gentamicin-resistant *S. aureus* (Figure 3A,B). In addition, we added the ROS scavenger N-acetyl-L-cysteine (NAC) and observed that the synergistic bactericidal effect was blocked with increasing NAC concentration, demonstrating the involvement of ROS in the AMP-mediated synergistic bactericidal effect (Figure 3C). The addition of AMP elevated the intracellular SOD activity and H_2_O_2_ levels, with no significant change in CAT activity (Figure 3D–F). Therefore, this cycle elevates endogenous ROS levels and increases the susceptibility to bacterial oxidative stress by generating H_2_O_2_ and decreasing the efficiency of ATP use [28].

### 2.4. The Synergistic Effect of AMP Is Extensive

**Figure 4:**
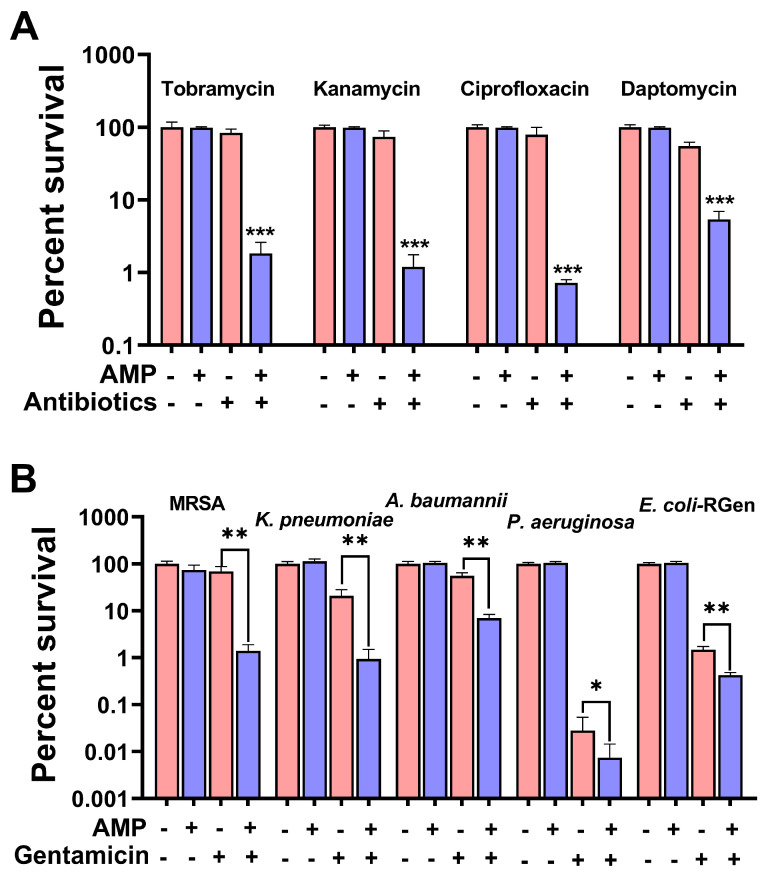
The combination therapy of AMP is extensive. (**A**) Percent survival of gentamicin-resistant *S. aureus* treated with 0.5 mM AMP and other antibiotics: tobramycin (400 μg/mL), kanamycin (2 mg/mL), ciprofloxacin (200 μg/mL), and daptomycin (200 μg/mL). (**B**) The synergistic bactericidal effects of combined treatment with AMP (0.5 mM) and gentamicin against MRSA252, *K. pneumoniae* ATCC10031, *A. baumannii* ATCC19606, *P. aeruginosa* ATCC27853, and gentamicin-resistant *E. coli*, with gentamicin concentrations of 800 µg/mL, 10 µg/mL, 5 µg/mL, 500 µg/mL, and 32 µg/mL, respectively. Results are displayed as mean ± SEM, and significant differences are identified (* *p* < 0.05, ** *p* < 0.01, *** *p* < 0.001) as determined using two-tailed Student’s *t*-test.

To determine whether this synergistic germicidal efficacy is universal, we conducted further studies. We observed that aminoglycosides (kanamycin and tobramycin), quinolones (ciprofloxacin), and cyclic lipopeptide antibiotics (daptomycin) had very strong synergistic bactericidal effects (Figure 4A). To explore whether AMP can also increase the susceptibility of other common bacterial pathogens to gentamicin, we first performed a minimum inhibitory concentration (MIC) determination on these strains. The results showed that MRSA252, *Klebsiella pneumoniae* ATCC10031, *Acinetobacter baumannii* ATCC19606, and *Pseudomonas aeruginosa* ATCC27853 were sensitive to gentamicin. Because of *E. coli*-RGen was selecting from the sequential propagation of *E. coli* K12 BW25113 with gentamicin, it developed a resistance to gentamicin, and had an MIC value of 80 µg/mL (Supplementary Figure S1). The combined AMP and gentamicin effect enhanced the killing efficacy against Gram-positive bacteria such as MRSA252 and Gram-negative bacteria such as *K. pneumoniae* ATCC10031, *A. baumannii* ATCC19606, *P. aeruginosa* ATCC27853, and gentamicin-resistant *E. coli*-RGen (Figure 4B). These results suggest that the synergistic effect of AMP can be generalized to a wide range of antibiotics and commonly resistant bacteria.

## 3. Discussion

AMP is a compound that occurs naturally in the body. It is often used in meat and poultry soups, or as a food additive for specific nutritional purposes [29]. People also use AMP as a supplement for athletic performance and cold sores, but there is no good evidence to support these uses. The limited number of human studies involving oral AMP have not indicated any side effects. However, doctors using AMP injections report that too-rapid intravenous administration or inadvertent administration of an intramuscular injection into a vein could cause life-threatening arrhythmias of the heart [30]. A previous study focused on people with an outbreak of shingles who were given injections of either a placebo or 100 mg of AMP three times a week for four weeks. The result showed that AMP could promote faster healing and reduce the duration of pain of the shingles [31]. However, there are few reports on common dosages of AMP in humans to date.

Numerous studies have demonstrated that the addition of specific exogenous substances can modulate bacterial susceptibility to antibiotics. Previous studies have found that the exogenous addition of uracil [15], fructose [32], glutamine [33], and pyruvate [34] has antibiotic bactericidal effects. These substances reprogram an antibiotic-resistant metabolome, such as the TCA or P cycle, into an antibiotic-sensitive metabolome, thereby increasing sensitivity [35]. Energy substances such as AMP, ADP, and ATP are involved in metabolic processes as direct energy donors or energy storage substances, indicating the importance of metabolism–energy substance associations in the reversal of bacterial drug resistance and the feasibility of metabolites for use as antibiotic adjuvants. However, few studies have been conducted on the resistance of Gram-positive bacteria to energy-producing substances, especially AMP.

In this study, we found that AMP was effective in enhancing the killing of Gram-positive bacteria using gentamicin by up to 100 folds. After identifying the synergistic bactericidal effects, we performed a series of mechanistic explorations. Energy and metabolism are highly correlated, and aminoglycoside antibiotics enter the cell via proton motive forces in the electron transport chain [36]. Therefore, the detection of Δψ and ΔpH revealed an increase in PMF. The ability of both the TCA-cycle-inhibitor malonic acid and the PMF-inhibitor CCCP to restore bacterial resistance also confirms that the synergistic effect of AMP and gentamicin is reflected in PMF, resulting in elevated intracellular antibiotic levels. A similar study demonstrated that AMP could potentiate the meropenem-mediated killing of carbapenem-resistant *A. baumannii*, and they speculated that it related to the PMF-dependent mechanism of AMP [35].

Researchers have found that increasing the production of endogenous ROS can impair the ability of bacteria to cope with oxidative stress [37,38]. Many studies have shown that antibiotics such as beta-lactams, aminoglycosides, and fluoroquinolones, regardless of their specific targets, induce oxidative stress, and thus participate in bacterial killing by antibiotic–ROS [39]. SOD catalyzes the disproportionation of superoxide anions to produce H_2_O_2_ and O_2_ [40]. CAT is the predominant H_2_O_2_ scavenging enzyme and plays an important role in the ROS scavenging system [41]. SOD activity increases, whereas CAT activity remains unchanged. This indicates that H_2_O_2_ cannot be decomposed in time after generation, and its accumulation in the cell results in an increase in ROS levels, a result that is consistent with our experimental results. When intracellular ROS levels are elevated, bacteria activate two oxidative stress pathways: reduced production and accelerated degradation [42]. This study revealed that AMP can enhance ROS production by elevating electron transfer and slowing degradation by modulating related enzyme systems, and that, under dual influence, bacteria become less viable. This was confirmed by the fact that the bacteria regained their resistance in the presence of the ROS scavenger NAC.

Interestingly, we found a decrease in intracellular ATP levels in the bacteria treated with AMP. The reason for this may be that ATP reduction is associated with a futile cycle, which reduces the efficiency of ATP production and use and increases the bacterial demand for ATP. Some studies found that ATP depletion may reduce the activity of the DNA repair system, impairing the cell’s ability to repair oxidative stress-induced damage, and may increase the production of more endogenous H_2_O_2_, decreasing the cell’s ability to cope with the additional oxidative stress [28,43], a result that is similar to those of our study. It has also been reported that adenosine potentiates antibiotic killing by generating PMF, which occurs independently of an ATP synthase while altering nucleotide metabolism, thus increasing ATP and GTP levels [44]. These studies will help to further explore the mechanism of AMP-enhanced antibiotic bactericidal effect. Generally, we believe that AMP acts as a precursor for the synthesis of ADP and ATP and participates in cellular life activities through the synthesis of energy substances. This study indicated that AMP can also be used as an antibiotic adjuvant.

This study still has some limitations, which need to be further improved in the future. 1. Limited in vivo studies: The research is primarily conducted in vitro. Including in vivo experiments would strengthen the clinical relevance of the findings. 2. Mechanism elucidation: While the study proposes mechanisms for AMP’s synergistic effect, further molecular details could be explored, such as specific interactions between AMP and cellular components. 3. Resistance development: The potential for bacteria to develop resistance to this combination therapy is not addressed. Long-term studies on the development of resistance would be valuable. 4. Dosage optimization: The study uses fixed concentrations of AMP and antibiotics. A more comprehensive dose-response analysis could help identify optimal ratios for different bacterial strains. 5. Safety considerations: The potential toxicity of AMP-antibiotic combinations to host cells is not thoroughly explored. This is crucial for assessing the clinical applicability of the approach. 6. Broader antibiotic panel: While the study tests several antibiotics, expanding the panel to include more diverse classes would provide a more comprehensive understanding of the synergy’s scope. 7. Clinical isolates: The study primarily uses laboratory strains. Including a wider range of clinical isolates would enhance the translational potential of the findings. 8. Biofilm studies: Given the importance of biofilms in antibiotic resistance, investigating the effect of AMP-antibiotic combinations on biofilm-associated bacteria would be valuable. 9. Pharmacokinetic considerations: The study does not address how the AMP-antibiotic combination might behave in a physiological context, including metabolism and excretion. 10. Comparative analysis: A comparison with other known antibiotic adjuvants would help contextualize the effectiveness of AMP.

## 4. Materials and Methods

### 4.1. Bacterial Strain and Culture Conditions

Gentamicin-resistant *S. aureus* (128-fold minimum inhibitory concentration (MIC), 320 µg/mL), gentamicin-resistant *E. coli* (64-fold minimum inhibitory concentration (MIC), 80 µg/mL), and methicillin-resistant *Staphylococcus aureus* (MRSA) 252 were obtained from a collection in our lab. As previously described [15], the *S. aureus* used in this experiment was obtained via an artificial passaging culture for gentamicin resistance and stored in our laboratory at −80 °C. The petri dishes containing the strains were stored at 4 °C. *Klebsiella pneumoniae* ATCC10031, *Acinetobacter baumannii* ATCC19606, and *Pseudomonas aeruginosa* ATCC27853 were purchased from the Guangdong Microbial Culture Collection Center (Guangzhou, China). These strains were cultured in Luria–Bertani (LB) broth medium. They reached saturation after 12–16 h of growth at 37 °C and 220 rpm with shaking.

### 4.2. Antibiotic Bactericidal Assays

The strain was activated overnight and collected, and its OD_600_ was adjusted to 0.2 with 5 mL of an M9 medium (containing 10 mM acetate, 1 mM MgSO_4_, and 100 µM CaCl_2_) after washing three times with sterile saline. Then, AMP and/or antibiotics and other related substances were added, and the control group was cultured at 37 °C and 220 rpm for 6 h. After a gradient dilution of a 100 μL bacterial solution, the 10 μL bacterial solution was placed in a petri dish and cultured in an incubator at 37 °C for 16 h. The number of colonies growing on the plate was calculated.

### 4.3. ΔpH and Δψ Measurement

The strain was activated overnight, and its OD_600_ was adjusted to 0.2 in an M9 medium. Then, AMP and/or gentamicin and other related substances were added, and the control group was cultured in 37 °C and 220 rpm for 6 h. A stained 198 μL bacterial solution with 2 μL 10 mM 5(6)-CFDA N-succinmidyl ester or DiBAC_4_(3) was added to 96-well plates and placed away from light for 1 h. A reading value of 485/515 nm was measured using an enzyme marker characterizing the magnitude of ΔpH and Δψ.

The measurement of intracellular gentamicin concentration was as follows. An ELISA test kit (JL17568) (JonIn, Shanghai, China) was used to detect the intracellular gentamicin concentration. Bacteria from the overnight cultures were collected as previously described [45], and the bacteria were resuspended in an M9 medium to OD_600_ = 1.0. Gentamicin and/or AMP were added and incubated for 6 h at 37 °C and 220 rpm. The cultures were collected following centrifugation at 8000 rpm, 37 °C, for 3 min and then washed three times with PBS (pH = 7.2) to ensure that any extracellular antibiotics had been completely removed. The bacteria were harvested and lysed via sonication (200 W total power with 50% output, 2 s pulse, and 3 s pause) on ice for 10 min. The supernatant was obtained following centrifugation at 12,000 rpm for 10min at 4 °C. Finally, the gentamicin concentration was determined using a diagnostic kit. The absorbance was measured at 450 nm using a plate reader (BioTek, Synergy HT, VT, USA).

### 4.4. ROS Measurement

ROS detection was performed as previously described [46]. The strain was activated overnight, and its OD_600_ was adjusted to 0.2 with 5 mL of an M9 medium after washing with normal saline. AMP (0.5 mM) or/and gentamicin (1.6 mg/mL) was added to the medium and incubated at 220 rpm at 37 °C for 6 h. Then, a 196 μL sample and 4 μL 2′,7′-dichlorofluorescein diacetate (Sigma-Aldrich, St. Louis, MO, USA) with a final concentration of 20 μM were added to 96-well plates and incubated at 37 °C for 1 h away from light. A reading value of 485/515 nm was measured using an enzyme marker.

### 4.5. ATP Measurement

Bac Titer-Glo microbial cell viability (Promega, Mannheim, Germany) was used to determine the amount of ATP. The strain was activated overnight, and its OD_600_ was adjusted to 0.2 with 5 mL of an M9 medium after washing with normal saline. AMP (0.5 mM) or/and gentamicin (1.6 mg/mL) was added to the culture medium and incubated at 37 °C and 220 rpm for 6 h. Then, a 50 μL sample and a 50 μL kit solution were added to a 96-well plate, and an enzyme meter was used to measure absorbance according to the manufacturer’s instructions.

### 4.6. H_2_O_2_ Measurement

H_2_O_2_ was measured using an H_2_O_2_ assay kit (Solarbio, Beijing, China). The activated bacteria were collected and diluted in an M9 basic medium with or without AMP (0.5 mM) with an OD_600_ value of 1.0. After incubation for 6 h, the cell precipitate was washed with PBS and resuspended. Incubation was then performed with lysin added, followed by sonication on ice for 20 min and then centrifugation at 12,000 rpm for 10 min to remove the precipitate. The supernatant was transferred into a new 1.5 mL tube, and the protein concentration was determined. Subsequently, the manufacturer’s instructions were followed, and finally, the absorbance was measured at 415 nm using the enzyme marker and calculated.

### 4.7. SOD Activity Measurement

A superoxide dismutase (SOD) kit (Solarbio, Beijing, China) was used for SOD activity determination. Briefly, activated bacterial fluids with OD_600_ = 1.0 were collected using an M9 medium. With or without AMP (0.5 mM), the bacteria were cultured at 37 °C for 6 h. Then, the bacteria were washed with PBS; resuspended; and removed by ultrasonic crushing on ice, lyase hydrolysis and precipitation, and centrifugation. After determining the protein content, the activity of superoxide dismutase was determined according to the manufacturer’s instructions, and the colorimetric reading was measured at 560 nm.

### 4.8. Catalase Activity Measurement

A catalase kit (Mlbio, Shanghai, China) was used for catalase activity determination. The bacteria were activated overnight, adjusted to OD_600_ = 1.0 with 20 mL of an M9 culture medium, cultured at 37 °C for 6 h with or without AMP (0.5 mM), and then collected by centrifugation. The bacteria were washed twice with PBS; re-suspended; and then removed by ultrasonic crushing on ice, lyase hydrolysis and precipitation, and centrifugation. After determining the protein content, the catalase activity was determined according to the manufacturer’s instructions, and the colorimetric reading was measured at 405 nm.

### 4.9. Determination of MIC

Minimum Inhibitory Concentration (MIC) determination was performed according to Clinical and Laboratory Standards Institute (CLSI) guidelines [47]. The strains were activated with Mueller–Hinton broth (MHB) medium overnight, transferred to fresh MHB medium at a 1:100 volume ratio and incubated until OD_600_ = 0.5, followed by a 100-fold dilution of the bacterial solution. MHB medium and antibiotics were added to 96-well plates, and the antibiotics were sequentially diluted 2-fold, followed by 10 μL of bacterial solution. The negative control group consisted of inoculated MHB medium only, and the MIC was observed after incubation at 37 °C for 12 h. The minimal antibiotic concentrations that displayed no visible growth were determined as MIC values.

## 5. Conclusions

**Figure 5:**
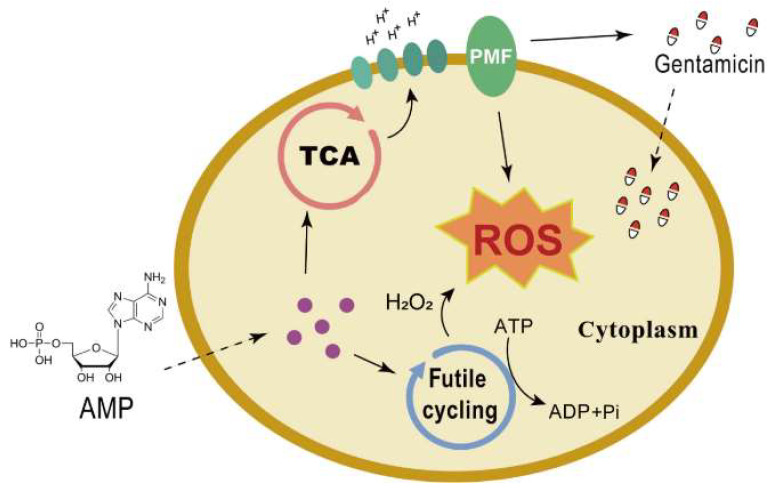
Schematic diagram of the synergistic bactericidal mechanism of action. AMP increases gentamicin uptake by the promotion of PMF and the generation of a futile cycle, while increasing bacterial ROS, leading to bacterial death.

In conclusion, the results of this study suggest that AMP promotes the killing effect of antibiotics on bacterial pathogens. Its mechanism of action includes increasing intracellular ROS levels to enhance bacterial oxidative stress and increasing PMF through the TCA cycle to mediate the entry of gentamicin into the cells (Figure 5). Our findings suggest a strategy to control bacterial resistance by adopting a low dose of antibiotics based on energy substance metabolites. This is important for future drug development and bacterial control through the regulation of energy substance levels to achieve the enhancement of multiple antibiotics for killing Gram-positive bacteria and other pathogens.

## Data Availability

The data are contained within the article.

## Supplementary Materials

The following supporting information can be downloaded at: https://www.mdpi.com/article/10.3390/ph17070933/s1, Figure S1. The MIC of different bacterial pathogens.
