# Supplementary figures and images for: Adenosine Monophosphate as a Metabolic Adjuvant Enhances Antibiotic Efficacy against Drug-Resistant Bacterial Pathogens
Source: Pharmaceuticals (Basel). 2024 Jul 11;17(7):933. doi: 10.3390/ph17070933 (PMC11280336; doi:10.3390/ph17070933)

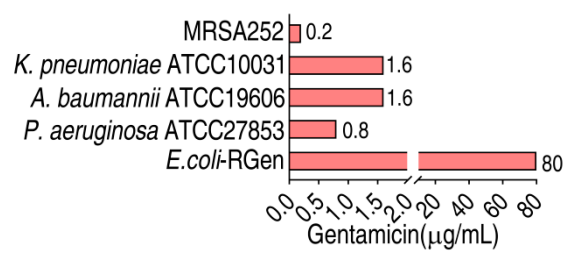

**Figure S1.** The MIC of different bacterial pathogens.

Supplement: Supplementary file 1 [file pharmaceuticals-17-00933-s001.zip › pharmaceuticals-3061247-supplementary.pdf]
